# Continuous preparation of carbon-nanotube-supported platinum catalysts in a flow reactor directly heated by electric current

**DOI:** 10.3762/bjoc.7.165

**Published:** 2011-10-14

**Authors:** Alicja Schlange, Antonio Rodolfo dos Santos, Ulrich Kunz, Thomas Turek

**Affiliations:** 1 Institute of Chemical Process Engineering, Clausthal University of Technology, Leibnizstr.17, D-38678 Clausthal-Zellerfeld, Germany

**Keywords:** carbon nanotubes, continuous catalyst synthesis, direct electrical heating, flow reactors, fuel cell platinum catalyst

## Abstract

In this contribution we present for the first time a continuous process for the production of highly active Pt catalysts supported by carbon nanotubes by use of an electrically heated tubular reactor. The synthesized catalysts show a high degree of dispersion and narrow distributions of cluster sizes. In comparison to catalysts synthesized by the conventional oil-bath method a significantly higher electrocatalytic activity was reached, which can be attributed to the higher metal loading and smaller and more uniformly distributed Pt particles on the carbon support. Our approach introduces a simple, time-saving and cost-efficient method for fuel cell catalyst preparation in a flow reactor which could be used at a large scale.

## Introduction

Batch processes represent the state of the art in catalyst preparation. One reason for employing this operation mode is that the yearly production rates can be rather small, comparable to pharmaceutical or fine chemical synthesis. With the advent of microreactors or minireactors continuous preparation methods have entered the organic chemist’s laboratory. This makes the small-scale preparation of products in a continuous operation mode attractive. On the one hand numerous organic reactions have been described in the flow mode [1–11]. On the other hand the preparation of catalysts in a continuously operated flow reactor is still a research field with only a few published results. Most of the work is concerned with the precipitation of hydroxides, oxides or other hardly soluble metal compounds [12].

Platinum nanoparticles supported on conductive carbon materials such as carbon black or carbon nanotubes (CNTs) are commonly used as oxygen reduction reaction (ORR) catalysts for direct methanol fuel cells (DMFCs). This kind of fuel cell has attracted great attention during recent years as a future power source for portable and stationary applications [13–15]. One of the advantages of methanol as fuel is its high energy density. Additionally it offers easy storage and transportation in comparison to hydrogen. At present, factors such as low power densities and high material costs, especially of the electrode, are the main challenges in widespread commercialization of DMFCs. Several research groups have shown that there is a clear correlation between the morphology of a carbon supported Pt catalyst and its electrochemical activities [16–18]. To overcome this challenge, further research on electrocatalyst development is a necessity. Generally, high metal content, small platinum cluster size, and uniform particle distribution over the support material are needed to enhance the electrochemical activity, resulting in higher power density values. It is known that the catalyst preparation method strongly influences the noble metal cluster size and its dispersion on the carbon and therefore the electrocatalytic activity [19].

DMFC electrocatalysts are mostly prepared in batch processes where an oil bath or heat exchangers act as the heat source. While using these traditional methods, hot spots can occur, mostly when a large volume of reaction mixture is used, hindering homogenous nucleation of platinum particles. Moreover, when using these preparation methods a longer time is needed to reduce the noble metal particles [20], resulting in an increase of the production costs. Additionally the cluster size and their distribution cannot be well controlled [21]. Furthermore, the amount of products is limited by the volume and size of the used reaction vessels.

In this contribution we demonstrate, for the first time, a simple and cost-effective method for the preparation of carbon-nanotube-supported Pt catalysts by using a continuously operated tubular flow reactor. The heating concept was realized by passing electrical current directly through the reactor wall. The experimental setup is not cost intensive, because all components used for the construction are standard laboratory equipment. Using the continuously operated tubular reactor, heating rates comparable to a microwave oven were achieved. Furthermore, the preparation technique is supposed to have a great potential also for the production of other metal/carbon supported catalysts.

### Electrocatalyst preparation methods

In recent years, different methods for the synthesis of carbon-supported Pt catalysts have been studied. Among these, three methods were mainly used:

1. **impregnation method**, based on the impregnation of platinum precursor salt on carbon support material followed by the reduction with a proper reducing agent (NaBH_4_, N_2_H_4_) or under a gaseous reducing environment (H_2_),
2. **microemulsion method**, based on a water–oil system where surfactant molecules are used for stabilization of nanoparticles, and
3. **colloidal method**, based on adsorption of platinum colloid on the surface of the carbon support material followed by the chemical reduction step.

The main advantage of the impregnation method is its simplicity in execution [22–23]. Nevertheless, catalysts prepared by impregnation show a broad cluster-size distribution and a large average cluster size resulting in lower electrocatalytic activity, as reported by [21]. The microemulsion method allows for better control of the nanoparticle size and distribution in comparison to the impregnation method. Disadvantages of this method are the high cost of the used surfactants and their time-consuming removal at the completion of the process [21], hindering the use of this method in a large-scale production. Therefore in recent years the colloidal method was often employed as the standard preparation technique for Pt deposition on carbon support. In this process (polyol reduction) ethylene glycol (EG) acts as both reducing agent and solvent for the Pt precursor. During the reduction step the solution of EG and Pt precursor salt is heated to 120–170 °C [24]. During this step EG is decomposed and generates the reducing species (CH_3_CHO, Equation 1). This species reduces the Pt ions to metallic Pt particles, as shown in Equation 2:

[1]
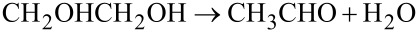


[2]
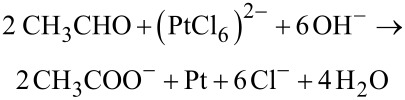


The main advantage of this polyol synthesis is that the acetate can also serve as a stabilizer for Pt colloids through the formation of chelate-type complexes through its carboxyl group [24]. The application of stabilization agents to protect Pt particles from agglomeration is not necessary. A precondition for a homogenous formation of nuclei during the polyol synthesis is the choice of a proper heating method. Conventional heating strategies, such as oil baths or heat exchangers, usually exhibit a heterogeneous temperature distribution including the possibility of hot spots. This can lead to temperature gradients in the reacting solution, resulting in poor dispersion of Pt on the carbon support. For this reason, in the past few years heating by means of microwave ovens was introduced. The microwave-assisted polyol synthesis method has many advantages over the conventional heating process [25–26]. It offers a more uniform environment for the nucleation and growth of metal particles [27], due to a rapid, homogeneous and effective heating. Moreover, fast heating rates can accelerate the reduction of the Pt precursor ions and the nucleation of the metal particles [27]. In consequence, the preparation of carbon-supported catalysts with smaller noble metal sizes and narrow size distributions is possible [21]. Amongst others, this attractive synthesis method for the production of CNT or carbon-black-supported Pt catalysts was successfully applied by several researchers [27–31]. It was possible to obtain highly dispersed Pt particles on the carbon support, resulting in an enhanced catalytic activity towards ORR, as described in [24,32–33].

The proposed microwave-heated polyol synthesis method was applied as a batch process only. Using our experimental setup it is possible to produce a Pt/CNT catalyst in a continuous polyol process. Heating rates generated during the reaction are comparable to a microwave oven. The apparatus does not require expensive temperature sensors as used in microwave heating systems. The costs for the described experimental setup are low compared to other heating equipment. In building our setup we incurred the following costs:

- reactor tube 20 EUR/m,
- two temperature controllers (with integrated power supply) 250 EUR each, and
- power supply – here a standard computer power supply was used (5V, 120A) 250 EUR.

Altogether, including the high diameter copper wires, some electronic components (MOSFET), standard tube connectors and the standard thermocouples, the costs for the materials were less than 1000 EUR. As remarked earlier, the setup consists solely of typical laboratory equipment such as NiCr-Ni thermocouples, Swagelok elements and stainless steel tubes. We think the presented approach is a flexible setup for the laboratory. Tubes in the range of 1/16'' to 1/4'' diameter made of stainless steel are appropriate starting materials for the reactor tubes.

Let us assume a reactor length of 50 cm with an inner diameter of 0.595 cm and an outer diameter of 0.635 cm, which is a standard 1/4'' tube. The specific measured resistance of the chosen stainless steel tube, made of 1.4404 (X2CrNiMo17-12-2), is 0.724 Ω·mm^2^·m^−1^ and thus the electrical resistance of this tube is 0.094 Ω. According to Ohm’s law, with a 5 V power supply a current of 53.2 A will develop inside the tube. This corresponds to a heating power of 265 W. This is sufficient to heat liquid reaction mixtures to reaction temperatures well above 100 °C in a short time if volumetric flow rates in the mL/min range are applied. Additional information on this aspect can be found in our previous publication [1]. The limitation of this concept is the availability of power supplies for high current since with tubes of larger diameter or shorter lengths the heating currents may reach more than 100 A. Up to this current rating the power supplies are low in price (less than 300 EUR) because these are produced for the large computer market. If different reactor lengths and diameters are to be tested it is recommended to purchase an adjustable low voltage/high current power supply. These are much more expensive, with prices ranging well beyond 1000 EUR.

## Results and Discussions

### Experimental setup

**Figure 1:**
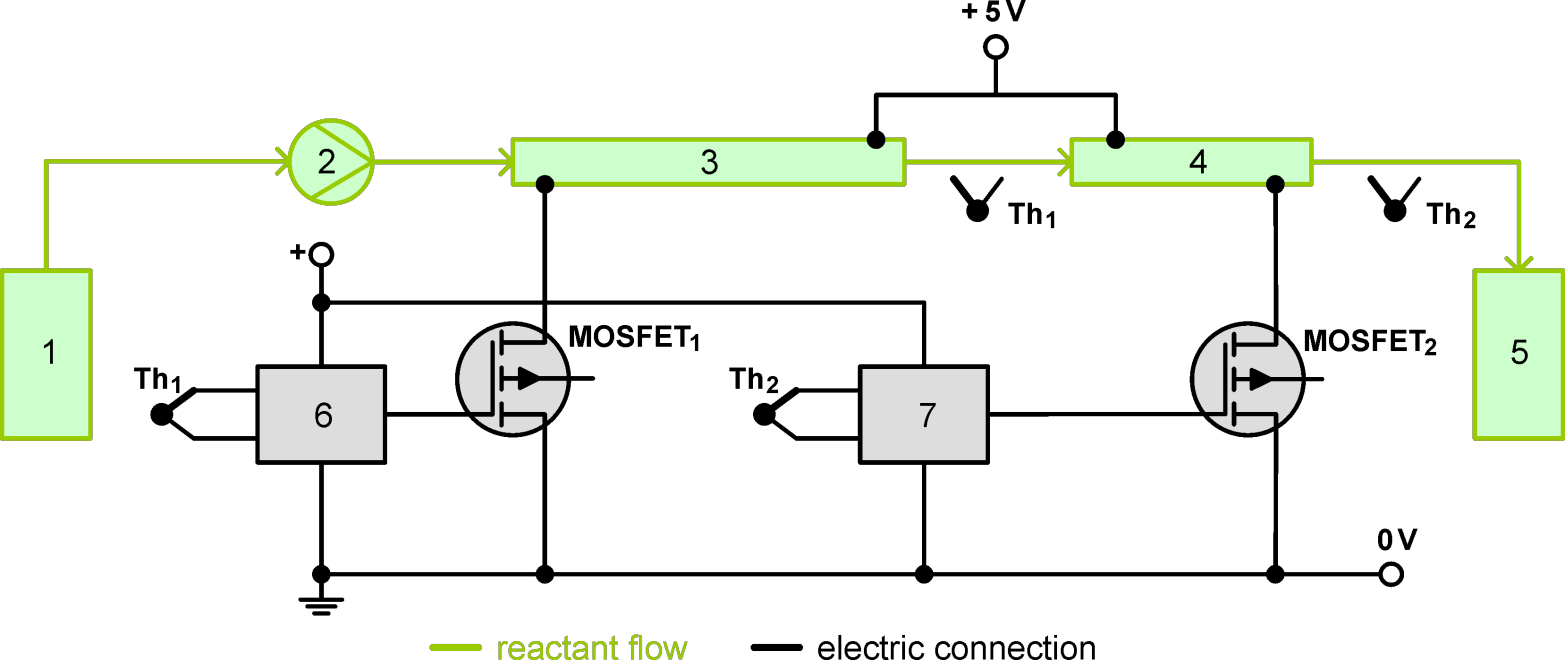
Experimental setup for catalyst synthesis in the tubular flow reactor; 1: Reaction mixture reservoir, 2: Peristaltic pump, 3: Preheating tube, 4: Heating tube, 5: Catalyst reservoir, 6: Temperature controller with thermocouple 1 (Th_1_), 7: Temperature controller with thermocouple 2 (Th_2_).

For the continuous preparation of CNT-supported catalysts an experimental setup as depicted in Figure 1 and similar to that described in our previous publication [1] was used. As seen in Figure 1 the reaction mixture of platinum precursor, EG and carbon nanotubes was pumped from the reservoir through two 1/8'' stainless steel tubes (3.18 mm × 0.56 mm) with a flow rate of 1 mL·min^−1^ by means of a peristaltic pump. In the first tube (length of 50 cm) the reaction mixture was preheated from room temperature up to a temperature of 140 °C within 90 s. Along the tube a linear temperature profile was established resulting in gentle preheating of the reaction mixture in a short time. In the second tube (length of 17 cm) this constant reaction temperature was maintained (residence time of 30 s). The connections between both tubes were made with Swagelok connectors.

**Figure 2:**
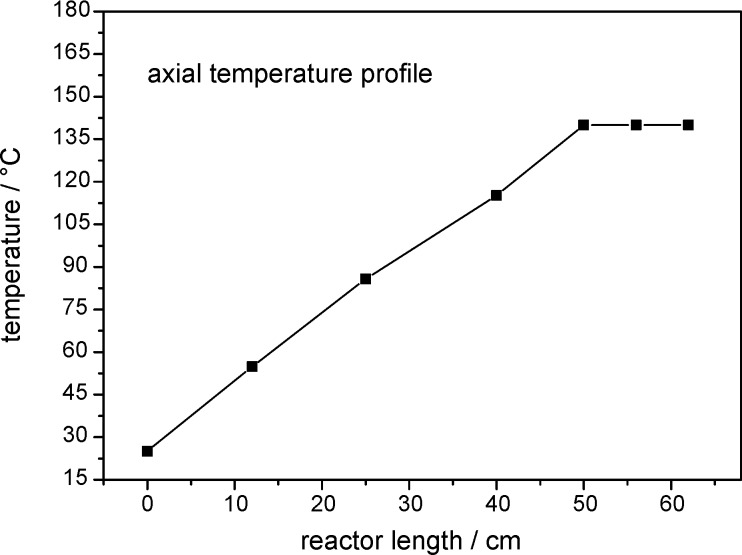
Measured temperature profile along the tubular reactor.

The heating concept is based on passing of the electric current through the reactor wall, which was delivered by a low voltage/high current power supply (5 V, 120 A). The advantages of this technique are the uniform heating across the whole surface area of the reactor without the occurrence of hot spots. In a previous work the temperature profile was measured with an infrared camera. This was published at a conference on infrared technology [34]. The temperature profile is linear since each volume element of the electrically heated tube produces the same amount of heat, which is caused by the fact that the average current along the tube is constant for a set heating rate. Overheating in the middle of the tube cannot occur and was not observed in the infrared measurements. In addition, we measured the temperature profile with thermocouples attached at different locations on the outer side of the reactor tubes. The measured axial temperature profile is depicted in Figure 2. This also demonstrates that the temperature profile is linear.

These reaction conditions promote the homogenous nucleation of platinum particles, resulting in a uniform metal distribution over the carbon support. For a precise temperature control NiCr–Ni thermocouples were installed at the outlets of both tubes. These were placed in the tube centre to adjust the temperature of the reaction mixture. All electrical connections within the heater supply lines were made of flexible, insulated 4 mm copper wires. At the outlet of the second tube the reaction mixture was continuously collected in a glass beaker and after cooling down and cleaning it was used for further cathode preparation. To investigate the reproducibility of the preparation method, three catalyst samples were taken at intervals of 25 min during the continuous reactor operation.

As a reference, an electrocatalyst was synthesized by a conventional method with traditional oil-bath heating. After preheating the oil bath to a temperature of 160 °C a 100 mL glass flask containing the reaction mixture was immersed. The temperature of the mixture was monitored during the chemical reduction with a Hg thermometer. A time of 10 min was needed to preheat the CNT/Pt-precursor/EG solution to the desired temperature of 140 °C. At this final temperature the reaction was performed for 3 h. Afterwards the product was cooled down to room temperature and cleaned several times with ethanol. Finally it was separated by means of a centrifuge at 4000 rpm and dried in an oven.

### Catalysts and support material characterization

**Figure 3:**
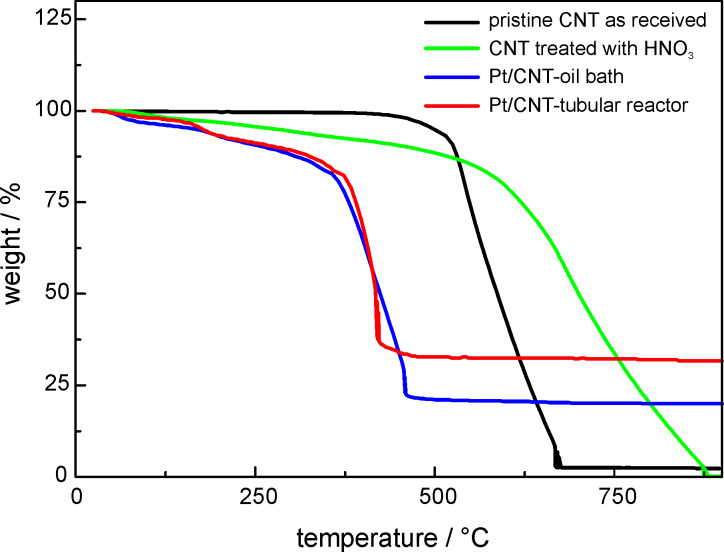
TGA weight loss curves for pristine CNT, HNO_3_ oxidized CNT, Pt/CNT-oil bath and Pt/CNT-tubular reactor samples.

Thermogravimetric analysis (TGA) curves for pristine CNT, oxidized CNT, Pt/CNT oil bath and one of the Pt/CNT tubular reactor samples are given in Figure 3. Differences regarding the oxidation behavior for the different samples can be clearly seen. Pristine nanotubes are thermally stable up to a temperature of 450 °C. The residue of 2.3% can be attributed to impurities remaining in the sample after the production process. In comparison to this result the oxidized CNTs starts to decompose at lower temperature. The thermal degradation of HNO_3_-treated CNTs in the temperature range between 150 to 350 °C is caused by the decomposition of the carboxylic groups attached to the surface during the nitric acid treatment. A weight loss of 4.69% in this temperature region reveals the successful functionalization of carbon nanotubes and formation of oxygen containing groups. For the electrocatalyst sample prepared in the tubular reactor a Pt loading of 31 wt % was estimated. In comparison, the reference catalyst synthesized by a conventional process exhibits a loading of 20 wt %. Differences in Pt amount between the calculated and the measured values after the chemical reduction were also reported by others. The reason for metal loss could be the repeated filtering and washing process as described in [21]. The observation that platinum loading is higher in the tubular reactor samples than in the oil-bath-prepared samples was confirmed by several samples. An explanation for this phenomenon is so far not available. One reason might be that in the oil-bath method we work in a system with back mixing whereas the continuously operated tubular reactor has no back-mixing effect.

**Figure 4:**
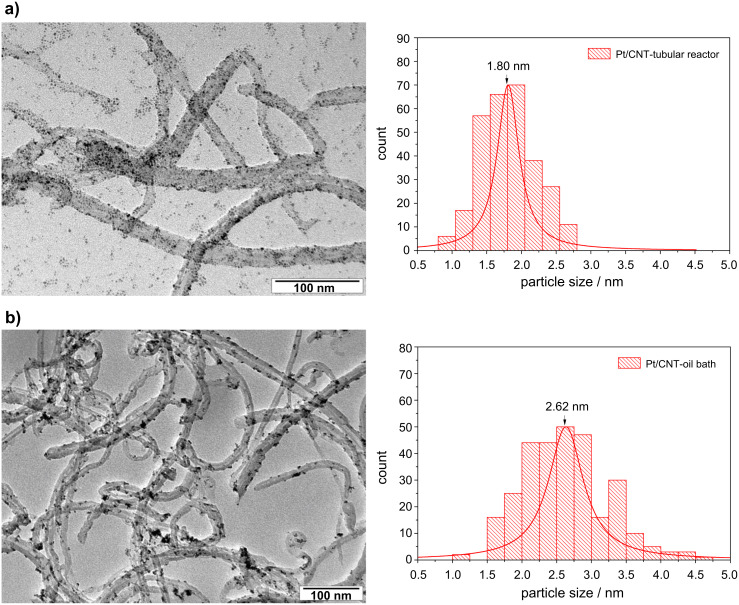
TEM micrographs of catalyst samples: a) Pt/CNT tubular reactor and b) Pt/CNT oil bath.

TEM micrographs of both catalyst samples are demonstrated in Figure 4. Cluster size histograms and the average cluster size are also shown. It can be clearly seen that for the catalyst prepared in the tubular reactor Pt particles are homogenously dispersed on carbon nanotubes. Particles range from 0.80 nm to 2.80 nm in diameter, with a mean value of 1.80 nm. In the case of the Pt/CNT catalyst synthesized in an oil bath the platinum particle sizes range from 1.00 nm to 4.75 nm resulting in an average size of 2.62 nm. For this catalyst sample some agglomeration of platinum particles was also observed as shown in Figure 4. The fact that some isolated platinum particles can be observed in the sample prepared in the tubular reactor can be attributed to the dispersion method employed during the TEM sample preparation.

These results reveal that the catalyst preparation method strongly influences the metal cluster size and distribution. Uniform and rapid heat transfer offered by the resistively heated tubular reactor accelerates the reduction of Pt ions, which results in the formation of smaller metal particles.

Metal dispersion is one of the most fundamental properties of supported metal catalysts [35]. To estimate the degree of metal dispersion for both our electrocatalyst samples, CO pulse chemisorption measurements were performed. Pulse chemisorption is useful to quantify the amount of active components on the surface of the supported catalyst. As described in [35] the extent of metal dispersion is defined as the fraction of metallic atoms present on the surface and therefore determines the catalytic properties. Platinum dispersion can be calculated from the following equation [36–37]:

[3]
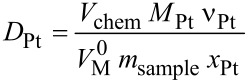



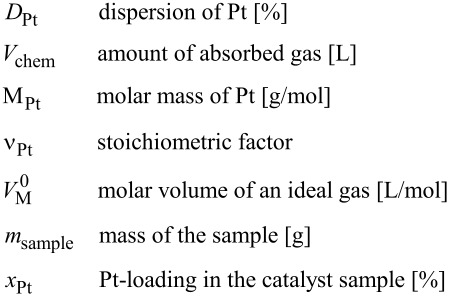


Using the CO chemisorption method a Pt dispersion of 16.49% for the Pt/CNT catalyst prepared in the tubular reactor was estimated compared to 12.65% for the reference catalyst. Dispersion values were measured for the same sample several times. In doing so the error of the method was found to be less than 5%. So the given values of 12.65 and 16.49% represent true differences between the two catalysts. As an additional reference, we have measured other catalyst samples indicating that the measuring method works accurately. We measured an industrial reference catalyst, 20 wt % Pt/C. The measured dispersion is 4.54%, which indicates that the sample prepared in the continuously operated tubular reactor has much higher dispersion.

**Figure 5:**
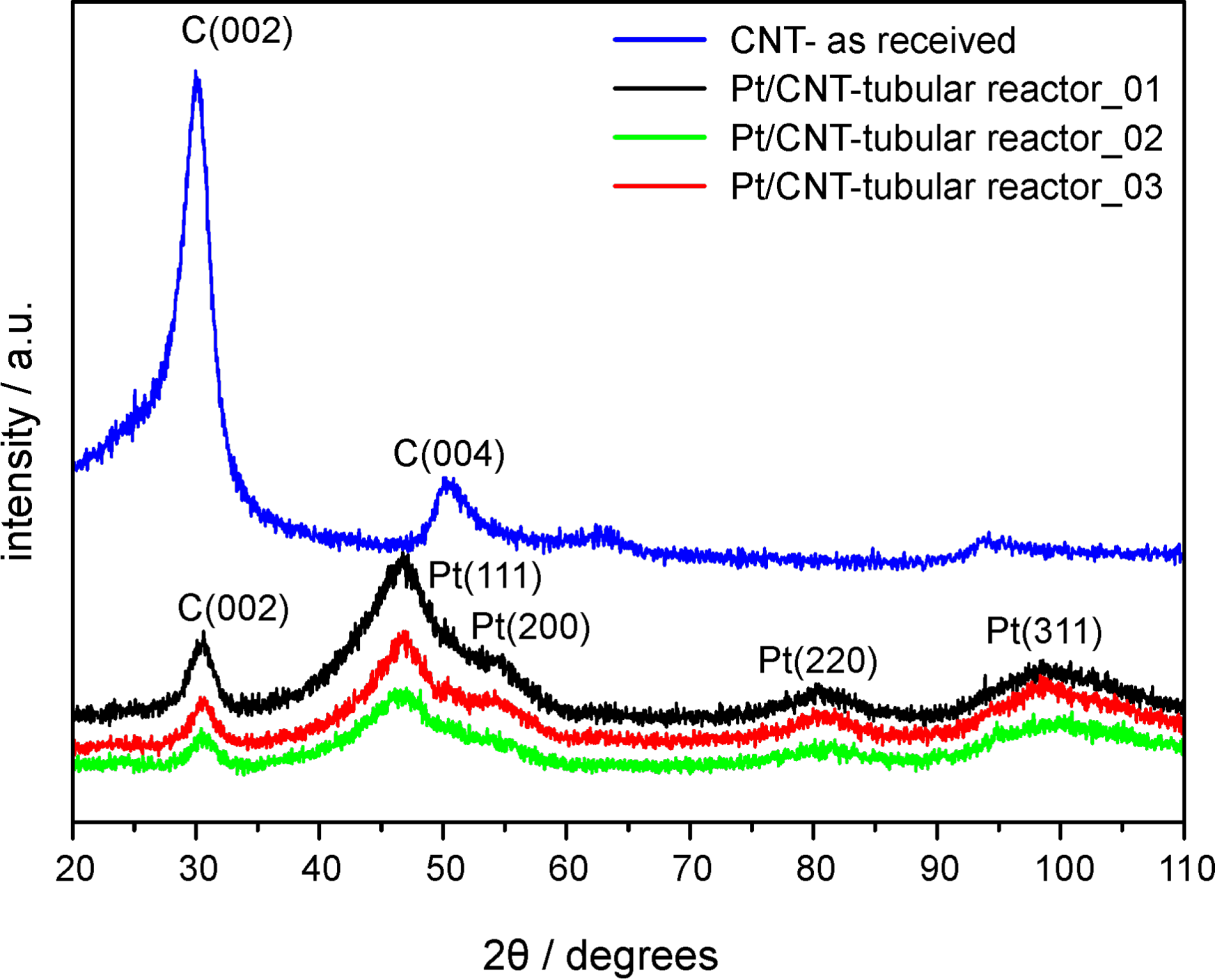
X-ray diffraction patterns for the as-received CNT and the three Pt/CNT samples taken at intervals of 25 min in the tubular reactor.

Powder X-ray diffraction analysis was performed to investigate the crystalline structure of the prepared catalysts and the support material. XRD spectra are given in Figure 5. For pristine carbon nanotubes the diffraction peaks at 30.0° and 50.4° can be attributed to the (002) and (004) planes of the hexagonal graphite structure.

For Pt/CNT catalysts prepared with the tubular reactor four characteristic peaks at 46.6°, 54.7°, 80.6° and 98.7° were found, which correspond to (111), (200), (220) and (311) reflection planes of the face-centered cubic (fcc) platinum, respectively. For these samples the diffraction spectra are very broad indicating small noble metal cluster sizes. The average Pt cluster size can be calculated from Pt (220) reflections with the Scherrer equation [38]

[4]
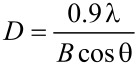


where λ is 0.178 and *B* is the full width at half maximum of the peak in radians. The Pt cluster sizes calculated for Pt/CNT catalysts prepared with the tubular reactor were measured as 1.77 nm, 1.74 nm and 1.77 nm. These results are in agreement with results determined by TEM analysis. For the reference Pt/CNT catalyst (not shown in Figure 5) the Pt cluster size calculated with the Scherrer equation was 2.31 nm.

### Electrochemical activity of prepared catalysts

**Figure 6:**
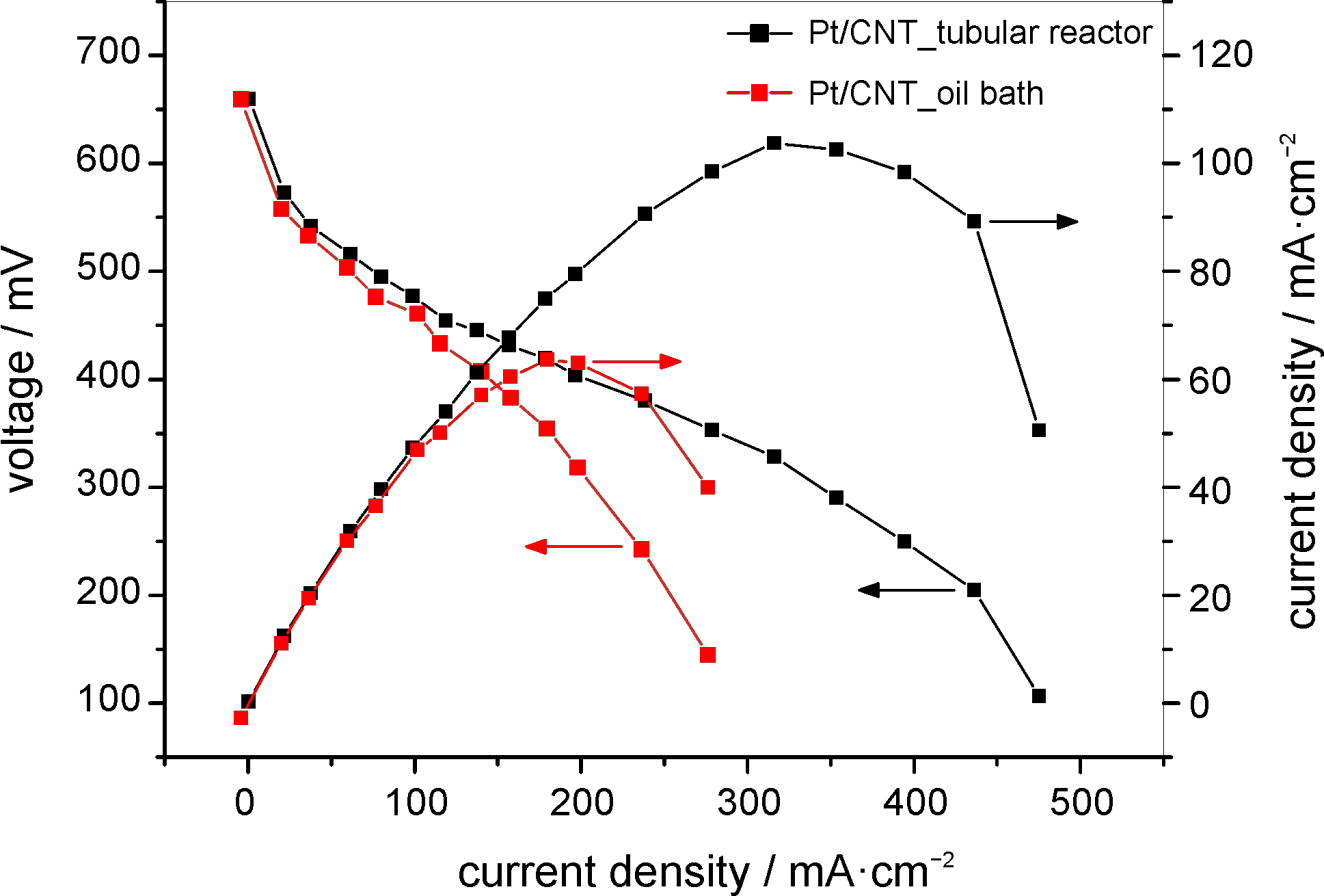
Comparison of performance in DMFC with Pt/CNT oil bath and Pt/CNT tubular reactor samples as cathode catalysts (Pt loading of 1 mg·cm^−2^ on each anode and cathode).

The single cell performances of the prepared Pt/CNT catalysts used as the cathode of a DMFC are shown in Figure 6. The measurements for both catalyst samples were performed under the same operating conditions (80 °C, 1 M MeOH at a flow rate of 5 mL·min^−1^, oxygen at a flow rate of 200 mL·min^−1^). In the fuel cell test the platinum loading in both experiments was the same. The differences of the Pt loading on the supports were taken into account. The higher loaded catalyst was applied in a smaller amount. Thus both electrodes had 1 mg Pt·cm^−2^.

Both samples show a nearly equal open circuit voltage (OCV) of 660 mV. The current densities at 0.4 V were found to be 203 mA·cm^−2^ for Pt/CNT (tubular reactor) and 139 mA·cm^−2^ for Pt/CNT (oil bath). The cell containing the electrocatalyst synthesized in the continuous process exhibits a maximum power density of 103 mW·cm^−2^ at 320 mA·cm^−2^. This means an increase in performance of 60% in comparison to the oil-bath sample (64 mW·cm^−2^ at 177 mA·cm^−2^). This improvement can be explained by the higher catalyst dispersion and the smaller platinum particles.

## Conclusion

The present work reveals that the preparation method is one of the most important factors determining the morphology and activity of Pt catalysts supported by carbon nanotubes. Short heating times and short reaction times are essential for the reduction of highly active platinum particles on nanotube supports. The proposed continuous preparation process offers simplicity in the production, a low cost experimental setup, and uniform reaction conditions due to a good temperature control. Therefore, the proposed preparation of Pt/CNT catalysts in a large-scale process is possible. In comparison to a reference catalyst prepared by a conventional method (oil bath) the samples synthesized in the tubular reactor show a more uniform distribution of the platinum cluster size (average particle size of 1.80 nm) without any agglomerates over the carbon support. The metal dispersion of the described catalyst (16.49%) is much higher than for the oil-bath sample (12.65%). DMFC performance tests reveal excellent catalytic activity of the catalyst prepared in the continuous process, resulting in a measured maximum power density of 103 mW·cm^−2^ in comparison to 64 mW·cm^−2^ for the reference sample. This improvement in fuel cell performance can be attributed to the smaller and homogenously dispersed Pt particles on the carbon nanotubes.

According to the obtained results, we can also state that the proposed continuous preparation technique by resistive heating may be very useful for the synthesis of other carbon-supported metal catalysts used in metal/air batteries or fuel cells, or even in other processes.

## Experimental

### 

#### Reagents

Carbon nanotubes (MWCNT, Baytubes®, C 150) with inner diameter of 2–6 nm and outer diameter of 5–20 nm were obtained from Bayer MaterialScience AG. Ethylene glycol (EG), HNO_3_, HCl and NaOH were purchased from Sigma-Aldrich and used as received. The platinum precursor (H_2_PtCl_6_·6H_2_O) was purchased from Merck.

#### Pretreatment of carbon nanotubes

The pristine nanotubes are inert and therefore have to be modified to create the anchoring sites for the Pt precursor. For this purpose 40 g of carbon nanotubes were first treated with 37 wt % HCl at 100 °C for 5 h to remove any impurities remaining after the production process. In the next step the purified nanotubes were refluxed with 1 L of concentrated nitric acid (65 wt %) for 5 h under a nitrogen atmosphere. After each acid treatment the product was cooled down, filtered, washed several times with deionised water to a desired pH value of 7 and finally dried in an oven at 60 °C for 24 h.

#### Preparation of electrocatalysts

Carbon nanotube supported Pt catalysts were synthesized by the polyol synthesis method (ethylene glycol mediated reaction). The procedure was as follows: 160 mg HNO_3_ treated carbon nanotubes were ultrasonically dispersed in 60 mL of ethylene glycole (EG). Then 265 mg of H_2_PtCl_6_·6H_2_O were dissolved in 20 mL EG and added dropwise into the prepared suspension of the carbon nanotubes. In the following step the pH value was adjusted to be above 10 through the use of 1 M NaOH. The resulting reaction mixture was used for electrocatalyst preparation, which was performed in a tubular reactor or by use of an oil bath (reference sample) as the heating source. After the reaction was completed, the resulting catalyst was cooled down to room temperature, washed with ethanol, then separated by means of a centrifuge at 4000 rpm and finally dried in a furnace at 60 °C under air flow.

#### Characterization methods

The crystalline structures of the support material and electrocatalysts were analyzed by X-ray diffraction (XRD) with a Siemens D5000/Kristalloflex diffractometer using Co Kα radiation (λ = 1.789 Å). The 2θ Bragg angles were scanned over a range from 20° to 110° with a step size of 0.02°. Transmission electron micrographs were obtained using a JEOL TEM 2100 with an accelerating voltage of 200 kV. Catalyst samples for TEM characterization were prepared by placing a drop of Pt/CNT suspension dispersed in ethanol on a carbon-coated copper (Cu) grid followed by drying at room temperature. The distribution of Pt particles over the carbon support was estimated with the LINCE 2.42e Software [39].

To investigate the thermal stability of nanotubes and to estimate the Pt loading of the catalyst samples, thermogravimetric analysis (TGA) was carried out. The measurements were performed with a Mettler TGA 860 thermo balance in air at a flow rate of 50 cm^3^·min^−1^ and a heating rate of 20 K·min^−1^ over a temperature range of 25–900 °C. The Pt loading in the sample was calculated from the last weight loss step at around 600 °C. The metal dispersion of the prepared electrocatalysts was estimated by means of a BELCAT-M (BEL Japan, Inc.) with 10 vol % CO/He gas at a flow rate of 15 mL·min^−1^.

#### Single cell test and electrode preparation

For electrocatalytic activity measurements a homemade DMFC test station equipped with high impedance potentiometer (Delta Elektronika SM3000) was used. The active area of the single cell with parallel flow field was 5 cm^2^. The platinum loading for both electrodes, anode and cathode, was 1 mg·cm^−2^, respectively. The fuel cell performance tests were carried out at 80 °C with pure oxygen as an oxidant (200 mL·min^−1^). 1 M MeOH solution at a flow rate of 5 mL·min^−1^ was supplied to the anode. The cathode layer was prepared using synthesized Pt/CNT catalysts, while the anode was fabricated using commercial Pt/Ru catalyst from BASF (40 wt % Pt, 20 wt % Ru) supported on Vulcan® XC-72R carbon. Catalyst coated membranes (CCM) were fabricated by spraying catalyst ink (water:catalyst:Nafion® ionomer (15 wt %) at a weight ratio of 9:1:1.175) on the Nafion® 117 membrane. After application of catalyst ink the membrane was hot-pressed at 120 °C at a pressure of 14 MPa for 3 min. The diffusion layers were prepared by coating a carbon cloth from Ballard (AvCarb 1071HCB) with a layer of 85 wt % carbon black (Ketjen Black EC 300J) and 15 wt % PTFE. A single DMFC test cell was finally assembled from diffusion layers, catalyst coated membrane, bipolar plates (material BMA5 from Eisenhuth GmbH & Co. KG) and Teflon gaskets.
